# Genomic Insights into Cardiomyopathies: A Comparative Cross-Species Review

**DOI:** 10.3390/vetsci4010019

**Published:** 2017-03-21

**Authors:** Siobhan Simpson, Paul Rutland, Catrin Sian Rutland

**Affiliations:** 1 Faculty of Medicine and Health Sciences, School of Veterinary Medicine and Science, The University of Nottingham, Sutton Bonington Campus, Loughborough LE12 5RD, UK; Siobhan.simpson@nottingham.ac.uk; 2 Great Ormond Street Institute of Child Health, University College London, 30 Guilford Street, London WC1N 1EH, UK; Paulrutland51@btinternet.com

**Keywords:** cardiomyopathy, human, canine, cross-species, cardiovascular disease, genetics

## Abstract

In the global human population, the leading cause of non-communicable death is cardiovascular disease. It is predicted that by 2030, deaths attributable to cardiovascular disease will have risen to over 20 million per year. This review compares the cardiomyopathies in both human and non-human animals and identifies the genetic associations for each disorder in each species/taxonomic group. Despite differences between species, advances in human medicine can be gained by utilising animal models of cardiac disease; likewise, gains can be made in animal medicine from human genomic insights. Advances could include undertaking regular clinical checks in individuals susceptible to cardiomyopathy, genetic testing prior to breeding, and careful administration of breeding programmes (in non-human animals), further development of treatment regimes, and drugs and diagnostic techniques.

## 1. Introduction

The cardiomyopathies (CMs) are a diverse group of cardiac disorders of the myocardium first described in the 1950s, usually exhibiting clinically unexplained hypertrophy or dilation of the ventricular walls and/or septum which can be either solely confined to the heart, or one part of a more generalised systemic disorder [1,2,3,4]. Cardiomyopathies are present in all populations and age groups and can lead to heart failure and death. Types of cardiomyopathy include: hypertrophic cardiomyopathy (HCM); dilated cardiomyopathy (DCM); restrictive cardiomyopathy (RCM); arrhythmogenic right ventricular cardiomyopathy (ARVC); and unclassified cardiomyopathies [5]. There is a range of mammalian species that can also develop the same cardiomyopathies either naturally or via induced disease processes. In addition, there are cardiomyopathies that are unique to a species/taxonomic group, such as great ape cardiomyopathy [6]. Although no genetic links have been published to date in relation to the great ape cardiomyopathy, collaborative work is presently underway throughout Europe under “The Ape Heart Project” [7]. Both natural and induced disease can act as models for other species, including humans, but naturally occurring cardiomyopathy (CM) in agricultural, working, and companion animals is also of clinical and financial concern in its own right.

The aetiology of the cardiomyopathies is complex, as causation can be both acquired and genetic in origin and often unknown. HCM is usually genetically inherited, with a prevalence of about 1:500 [5]. DCM is genetic in about one-third of cases [8] and can also be caused by external factors such as alcohol, toxins, drug abuse, viral infections, and pregnancy. Many (25%) of the mutations associated with this form are in the large titin gene [9]. RCM is rare genetically and can be caused as a result of connective tissue disorders, sarcoidosis, amyloidosis and haemochromatosis, eosinophilic heart disease, or as a result of radiation treatment [10], but has also been linked to several genetic mutations [11,12]. ARVC has been linked to several genes, but mutations in genes affecting the desmosomes occur most frequently within this disorder. Linkage of CM to a genetic locus was first reported in 1989 [13], and in 1990 the first causative gene mutation (in cardiac beta (β)-myosin heavy chain; *MYH7/βMHC*) was reported [14]. The genetics of the CMs is complex due to both phenotype and genotype heterogeneity over a wide range of severities. Symptoms can vary from none to very extreme, and risk of death may vary from almost zero with a normal lifespan to early infant death and clinical progression, and from no measurable progression to rapid and early symptoms. To complicate matters further, the transmission is often autosomally dominant but with variable penetrance, and there are affected genes on the X chromosome (recessive inheritance) and also within the mitochondrial genome which are maternally inherited. This review gives an oversight into what has been published for human and non-human animals to date. The search terms throughout included cardiomyopathy alongside a species name using both the Latin and common names. 

## 2. Hypertrophic Cardiomyopathy (HCM)

In the 2014 European Society of Cardiology (ESC) guidelines on diagnosis and management of hypertrophic cardiomyopathy [15], the disorder is defined by “the presence of increased left ventricular wall thickness that is not solely explained by abnormal loading conditions”. It is characterised by left ventricular hypertrophy (without dilation) and abnormally large and misaligned myocytes localised to the interventricular septum; many patients develop dynamic obstruction to left ventricular outflow and increased fibrosis which leads to diastolic heart failure [5,16]. HCM is the most common form of CM, which has a reported prevalence of 0.02%–0.23% in adults [15,16]. There have been HCM-associated mutations in over 20 genes associated with the sarcomere, myofilament, Z-discs, and calcium handling in humans [17].

Most of the HCM mutations (up to 80% [18]) come from the beta myosin heavy chain (*MYH7* 25%–40%) and cardiac myosin binding protein C genes (*MYBPC3*, 25%–40%), which are genes of the sarcomere/myofilament proteins that generate the force for myocyte contraction. The cardiac troponin T genes *TNNT2* and *TNNi3* contribute a further 10% more cases and it is well established that genes of the Z-disc and calcium-handling genes, amongst many others, contribute less commonly to the causes of CM [16,18].

In addition to its occurrence in people, HCM occurs naturally in both cats [19] and dogs [20,21]. It is more common in cats than dogs, accounting for <1% of canine cardiovascular diagnoses [20], but it has been diagnosed in up to 14.6% of apparently healthy cats [22]. Feline hypertrophic cardiomyopathy appears to have a breed predisposition, as does canine DCM and is, reportedly, the most common cardiac disorder in cats and has shown remarkable similarities to human HCM [19]. The familial predisposition is most obvious in Maine Coon, Ragdoll, and British and American Shorthair breeds [23,24,25]. Separate genetic mutations have been identified as causal of HCM in the Maine Coon and Ragdoll breeds within the *MYBPC3* gene [24,26] but there are also affected individuals within the Maine Coon breed lacking the mutation, indicating that there are additional causes of the disease to be established [27]. *MYBPC3* is a gene with mutations associated with human HCM [28]; thus, in these cases, feline and human HCM may act as relevant models for each other. This is important, considering that an estimated 35% of human cases are caused by *MYBPC3* mutations [16]. Higher concentrations of cTnI have also been observed in some breeds of cats with HCM [25,29], but no mutations have been observed in that gene in Maine Coons or British Shorthairs [25], whereas mutations in this gene have been observed in humans. Pigs also appear to have a heritable form of HCM, but no specific genetic associations have been discovered to date [30]. 

There are fewer available induced models of HCM compared to DCM. The primary model type being transgenic models, generally based on genes associated with human HCM in order to create the HCM phenotype. Transgenic and naturally occurring strains of mice [31], rabbits [32], and hamsters [33] have been used as models of HCM. Some strains of hamsters—including BIO14.6 and TO-2—develop HCM and DCM showing mutations in the delta-sarcoglycan gene, however, the phenotype differs a little from humans in that it shows augmented necrosis [34,35]. 

## 3. Dilated Cardiomyopathy (DCM)

Dilated cardiomyopathy is defined by left ventricular chamber enlargement and systolic dysfunction. Enlargement of the cavity and thinning and weakening of the walls of the left ventricle generally progresses to the right ventricle over time. The inability to pump properly leads to heart failure and clot formation in the heart in some species [36]. DCM is estimated to have a prevalence of 0.036% in humans but this is thought to be an underestimate and, in common with the other cardiomyopathies, the incidence is increasing with better testing and recognition [37,38,39]. To date, there have been mutations in over 50 genes associated with human DCM [40]. 

Naturally occurring spontaneous DCM exists in several species including dogs [41], cats [42], cattle [43,44,45], turkeys [46,47], and chickens [48]. Feline DCM is frequently associated with a lack of dietary taurine, and since this discovery commercial pet foods are now supplemented with taurine. Feline DCM is now rare [49,50]. Despite this, there are instances of feline DCM that are not related to taurine deficiency [42,50]. These instances are currently unexplained, but it is possible that, as with other species, the underlying cause of these cases is genetic. No genetic loci have been associated with feline DCM, but evidence for a genetic involvement in the development of feline DCM has been demonstrated [51].

Naturally occurring canine DCM is common—10% of dogs that died from cardiac disease within the insured Swedish dog population had a diagnosis of DCM [20]. Whilst canine DCM is common, there are some breeds that are particularly susceptible to developing the disease [20]. There are thought to be several sub-types of canine DCM which may reflect those seen in human DCM [52]. An example of this is juvenile DCM that occurs in Portuguese water dogs, which may reflect childhood DCM in humans [53,54]. Despite similarities with human DCM, and many studies testing for genetic associations with canine DCM, there have only been 10 loci associated with adult-onset canine DCM [55,56,57,58,59], and recently reviewed by Simpson et al. [52]. There has been an additional locus identified as associated with juvenile-onset canine DCM [60].

DCM in cattle is reported to particularly affect Red Holstein and Simmentaler X Red Holstein individuals [43,44]. It is possible that individuals from other breeds of cattle develop DCM, but within the commercial dairy and beef industries any such disease is likely to be rapidly selected against as it would have an impact on production. DCM in cattle was demonstrated to be inherited in an autosomal recessive pattern [61]. A nonsense mutation in the outer mitochondrial membrane lipid metabolism regulator (*OPA3*) gene has subsequently been demonstrated to be responsible for these incidences of bovine DCM [44].

DCM in turkeys has been associated with unusually low molecular weight cardiac troponin T due to the splicing out of exon 8 [46]. Exon 8 is not normally spliced out of avian and mammalian cardiac troponin T, thus it is considered to be abnormal with probable functional implications contributing to the development of turkey DCM [46]. Exon 8 is also spliced out of cardiac troponin T in wild turkeys [46] and thus likely contributed to the death of the wild turkey [47]. It is of interest that cardiac troponin T is also implicated in humans [62,63] and a mouse model [31]. Chicken DCM has been reported as a naturally occurring disease [48], but also of interest are the *in ovo* chicken models showing that knockdown of the myosins—specifically, embryonic myosin heavy chain (*eMHC*)—results in DCM [64], as does alpha myosin heavy chain (αMHC/*MYH6*) [65] which also affects humans [66,67]. 

DCM-induced animal models are frequently used in order to understand not only the genetic factors but also the structural and functional aspects of the disease. Several inbred strains of hamster develop DCM [35] and a number of methods are used in different species to induce disease using chemical and mechanical induction, including pacing [68,69,70,71]. 

## 4. Restrictive Cardiomyopathy (RCM)

Restrictive cardiomyopathy results in dilated atria and stiffening of the ventricles, which leads to restrictive filling and reduced diastolic volume, inefficient pumping, and heart and valve failure. Hypertrophy is typically absent and systolic function is usually unaffected [71]. RCM in children is rare: it accounts for only 2%–5% of childhood cardiomyopathies [72]. Although the incidence of RC is rare, it has a poor prognosis, with 30% of affected patients dying within 5 years of diagnosis [5]. Although RCM is much rarer than DCM, HCM, and ARVC, mutations in several genes have been associated with RCM, including beta myosin heavy chain (*MYH7*) and troponin I [12,73,74]. The RCM-causing mutation was identified in 19 patients (60%). Mutated genes have also been implicated in a number of idiopathic RCM cases. In a recent large cohort, the number of affected individuals showing a genetic change numbered between one and four patients for each gene investigated, *MYH7* (four patients), desmin and filamin C (*DES* and *FLNC*; three patients each), *MYBPC3* and lamin A (*LMNA*; two patients each), titin-cap, troponin I3 cardiac type, troponin T2 cardiac type, tropomyosin 1 (alpha), and lysosomal associated membrane protein 2 (*TCAP*, *TNNI3*, *TNNT2*, *TPM1*, and *LAMP2*; one patient each) [75,76].

RCM has recently been shown to naturally affect cats [77,78]. There have not been any loci associated with feline RCM and from the initial report it may be difficult to identify genetic loci as there does not appear to be familial inheritance because, in this study, individuals were from a range of breeds and were not known to have been related [77]. A mouse model expressing a missense mutation (R193H) in troponin I3 cardiac type (*CTnI*) has been developed [79]. Mice affected by sickle cell anaemia have recently shown symptoms of RCM [80] and this link has also been confirmed in humans [81]. There is some discussion as to whether this is a unique form of cardiomyopathy or RCM, as it is characterised by diastolic dysfunction, left atrial dilation, and normal systolic function [81].

## 5. Arrhythmogenic Right Ventricular Cardiomyopathy (ARVC)

Arrhythmogenic right ventricular cardiomyopathy (ARVC also called ARVD) is characterised by fibro fatty replacement of the right ventricular myocardium [82]. As the proteins forming the heart scaffolding become defective, muscle cell death occurs and muscle tissue is replaced with fatty and fibrous tissue. The heart walls become thinner and pumping efficiency and cardiac rhythm are affected, leading to heart failure [82]. In humans, ARVC has a reported prevalence of 0.02%–0.10% in the general population and is associated with other disorders such as amyloidosis [18,83]. There have been mutations in several genes identified as associated with human ARVC, in particular, mutations in the five desmosomal genes—desmoplakin, plakophilin 2, desmoglein 2, desmocollin 2, and junction plakoglobin (*DSP*, *PKP2*, *DSG2*, *DSC2*, and *JUP*) [84]. It is thought that around 50% of symptomatic humans have a mutation in one of these five cardiac desmosome genes. Other non-desmosomal genes have also been implicated including transforming growth factor beta 3, transmembrane protein 43, desmin, lamin A, titin, phospholamban, and catenin alpha 3 (*TGFB3*, *TMEM43*, *DES*, *LMNA*, *TTN*, *PLN*, and *CTNNA3*) [85]. In addition, ryanodine receptor 2 (*RYR2*) has been implicated in both human ARVC [86] and in a chronic anthracycline-induced cardiomyopathy in mice [87]. Numerous mutations have been described in relation to causing ARVC including the arrhythmogenic right ventricular dysplasia genes *ARVD3* (14q12– q22) [88], *ARVD4* (2q32.1– q32.3) [89], *ARVD*6 (10p14– p12) [90,91], and *ARVD7/ARVC7* (10q22.3) [92]. 

ARVC naturally affects dogs but has only been widely reported in the Boxer breed [93] and has been suggested as a model of human ARVC. There has been a mutation in the striatin (*STRN*) gene associated with ARVC in the Boxer dog [94]; it is of interest that the same gene has also been associated with DCM in the Boxer [57]. Striatin has not yet been implicated in human ARVC, but could be an area of interest to investigate. There is a syndrome in cattle that has a cardiac element to it that resembles that observed in humans where the cardiac element is ARVC. A mutation in the nuclear factor kappa B subunit 1 (*NFKB1*) gene is associated with this syndrome [95]. It is of interest that a functional polymorphism of *NFKB1* has also been linked to human DCM [96]. Despite identifying ARVC in cats over 15 years ago [97], and more recently in horses [98,99], there are no reports of genetic associations with feline or equine ARVC in the literature to our knowledge.

## 6. Mitochondrial, X Linked, and Peripartum Cardiomyopathies

It has been suggested that ~5% of DCMs are X-linked [100]. The X-linked cardiomyopathies are often associated with systemic general disorders such as Fabry’s disease, Barth syndrome, and Duchenne and Becker muscular dystrophy, and are most commonly associated with DCM and HCM [5,101,102,103,104]. Most of the work is presently in humans and very frequently on families, but the main proteins mutated are tafazzin (G4.5), emerin, lysosome-associated membrane protein 2, XK membrane transport protein, and dystrophin [104,105,106,107,108]. 

It is likely that mitochondrial genes are often associated with HCM and DCM because the heart is a high user of cellular energy provided by mitochondria. tRNA genes are associated with myoclonic epilepsy with ragged red fibres (MELAS) and mitochondrial encephalopathy with lactic acidosis and stroke-like episodes (MERRF) syndromes, and inheritance is maternally inherited [109]. This category often includes cardiomyopathy caused as a result of general systemic disorders where symptoms are not wholly associated with the heart [5]. HLA-DRB1*0901 allele has also been associated with a number of DCM patients, which suggested that mitochondrial ADP/ATP plays a large role in appropriate functionality of the heart [110]. It has also been shown that mitochondrial mutations are associated with changes in cardiomyopathy forms. The Mt8348A→G mutation in the mitochondrial tRNA(Lys) gene has shown a phenotypic alteration from hypertrophic to severe dilated cardiomyopathy [111], but so far only one case has been shown and therefore more patients need to be analysed. The role of mitochondria in the heart is essential, and drug-induced murine models causing mitochondrial damage result in cardiovascular arrhythmias and cardiomyopathy [112]. Humans are not the only species to have mitochondrial gene mutations associated with CM. Mutations in *PDK4*, a mitochondrial gene, are associated with Doberman pinscher DCM in the dog [58], therefore, it is essential that new studies also investigate mitochondrial genes.

Peri- or postpartum cardiomyopathy (PPCM) has been described under a number of conditions, but is defined by the European Society of Cardiology as the “development of heart failure toward the end of pregnancy or in the months following delivery” [113]. This has been observed in many species including human, canine, and bovine cases [113,114,115]. Much of the literature indicates that peripartum cardiomyopathy might be better referred to as DCM that is initiated during pregnancy or soon thereafter, but discussions are still ongoing as to whether this is an accurate portrayal [116]. In the case of peripartum CM, it has been shown that oxidative stress and prolactin play roles in the disease in humans and mouse models [116,117]. Of particular interest are the few human genetic studies that have been carried out to date, all of which associate PPCM with DCM causing mutations in *TNNC1* and *TTN* in humans [118,119,120]. The complex nature of this cardiomyopathy, potential overlaps and links to DCM, and the frequently observed hypertension, preeclampsia, and altered hormone levels make this a difficult CM to investigate, and more work needs to be undertaken, not only in humans but in other species too. 

## 7. Conclusions

Common genetic pathways could exist among cardiomyopathies and among species. As discussed above, there are multiple genes where differing mutations within each gene can cause different CMs in humans. Equally, there are many examples where genes have been implied in humans but not non-human animals—and vice versa—such as the striatin gene mutations, which are associated with both ARVC and DCM in Boxer dogs [57]. It is also clear that different mutations in the same gene can cause different types of CM. Both DCM and HCM in humans have been linked to *TTN*, *MYH6*, *MYH7*, *MYBPC3*, *TNNT2*, *TNNI3*, *TPM1*, *ACTC/ACTC1*, *TNNC1*, *ACTN2*, *ANKDR1*, *CSRP3*, *LDB3*, *TCAP*, *VCL*, *PLN*, and *RYR2* [17,52]; likewise, mutations in *MYH7* and *TNNI3* can cause RCM, DCM, and HCM [40,121]. *RYR2* has been implicated in ARVC, DCM, and HCM [86,121], and the ARVC genes *DSP*, *PKP2*, *DSG2*, *DSC2*, and *JUP* have all been associated to DCM [17]. Upon tabulation of 50 genes causing HCM, DCM, and ARVC, it was shown that the degree of heterogeneity in contributing genes is considerable [18]. In their review of 50 genes causing HCM, DCM, and ARVC, only 7 genes had changes unique to HCM and 15 genes had changes unique to DCM, while changes in 33 other genes contributed to both disorders. Similarly, ARVC currently has 3 genes contributing solely to ARVC and a further 7 genes associated with DCM, HCM, and ARVC. Most of the mutations causing ARVC are found in the genes *PKP2* and in the desmosomal cadherins, and these genes are also associated with DCM. Many of the CM-associated mutations are present in the structural genes, which are phylogenetically highly conserved; therefore, the frequent similarities in mutations/genes affected between humans and animals are not surprising. This further supports the use of multiple species investigations when looking at the differing genotypes in order to understand the cardiomyopathies. A summary of all genes associated with naturally occurring CMs in each species has been compiled in Table A1. 

With both genotype and phenotype overlapping greatly between the different cardiomyopathies, it is likely that, in the future, diagnosis will rely greatly on next-generation sequencing (NGS) technology and genome-wide association studies. Lessons may be learned about the genetic causes of CMs using information from these studies, also. Many of the studies, both human and animal, have previously relied on familial studies or cohorts with relatively small numbers. Over the years, study sizes have increased. As more advanced technology is more readily available, and at a lower cost, the candidate gene approach is being replaced with larger sequencing studies on larger cohorts. Examples of this are already observed throughout the literature in this review, but national and international endeavours such as the 100,000 genome project [122] are utilising the power of mutation and disorder detection, not only in common disorders but also in rare CMs. Large-scale studies such as these will frequently have to be supported by cohort studies. A number of papers have suggested that mutations in specific genes are not exclusively involved with particular CMs in individual species or breeds or animals, rather that different mutations in the same genes could well cause differing CMs [123,124,125]. Critical analysis of the sample sizes should be carried out before genes and/or mutations are ruled out. The genetic heterogeneity of CM genes can be indicated with the knowledge that a recent diagnostic NGS panel for CM diagnosis has 104 genes and candidate genes designed from research papers in the field [126]. Testing not only humans, but animals too, will not only aid in diagnosis and prognosis, but potentially assist with understanding epidemiology of the disorder. Genetic testing will also assist with healthcare options and treatment plans even prior to clinical symptoms of the CM, and aid in the reduction of affected animals within breeds. Although many of the cardiomyopathies characterised to date are single gene disorders, there is increasing evidence that multiple gene associations can contribute towards this disease. This has been evidenced in dogs [124,125], but more research needs to be undertaken in order to understand the situation for each CM type in each species. 

Cardiomyopathies are complex cardiovascular disorders, but advances in genetic detection are important not only to humans but also in animals, as models of the human condition, but also in order to advance non-human healthcare and breeding programmes. Targeted healthcare, diagnosis and prognosis are essential for cardiomyopathy patients, and further insights into the genetic causes are essential.

## Appendix A

**Table A1:** Genes associated with naturally occurring cardiomyopathies in human, canine, feline, bovine, and turkey.

Gene	DCM				HCM		ARVC			RCM	References
	Human	Canine	Bovine	Turkey	Human	Feline	Human	Canine	Bovine	Human	
*ABCC9*	Y										[127]
ATP Binding Cassette Subfamily C Member 9											
*ACTC1*	Y				Y						[128,129]
Actin, Alpha, Cardiac Muscle 1											
*ACTN2*	Y				Y						[130,131]
Actinin Alpha 2											
*ANKRD1*	Y				Y						[132,133]
Ankyrin Repeat Domain 1											
*ARGHAP8*		Y									[58]
Member of the RhoA activating protein family											
*ARVD3*							Y				[88]
Arrhythmogenic Right Ventricular Dysplasia 3											
*ARVD4*							Y				[89]
Arrhythmogenic Right Ventricular Dysplasia 4											
*ARVD6*							Y				[90,91]
Arrhythmogenic Right Ventricular Dysplasia 6											
*BAG3*	Y										[134,135,136]
BCL2 Associated Athanogene 3											
*CALR3*					Y						[137]
Calreticulin 3											
*CASQ2*					Y						[137]
Calsequestrin 2											
*CAV3*	Y										[138]
Caveolin 3											
*CHRM2*	Y										[139]
Cholinergic Receptor Muscarinic 2											
*CRYAB*	Y										[140]
Crystallin Alpha B											
*CSRP3*	Y				Y						[131,141]
Cysteine and Glycine Rich Protein 3											
*CTF1*	Y										[142]
Cardiotrophin 1											
*CTNNA3*							Y				[143]
Catenin Alpha 3											
*DES*	Y						Y			Y	[75,144,145,146,147]
Desmin											
*DMD*	Y	Y									[59,148,149]
Dystrophin											
*DNAJC19*	Y										[150]
DnaJ Heat Shock Protein Family (Hsp40) Member C19											
*DOLK*	Y										[151]
Dolichol Kinase											
*DSC2*	Y						Y				[84,152]
Desmocollin 2											
*DSG2*	Y						Y				[153,154,155]
Desmoglein 2											
*DSP*	Y						Y				[156,157]
Desmoplakin											
*EMD*	Y										[158]
Emerin											
*EYA4*	Y										[159]
EYA Transcriptional Coactivator and Phosphatase 4											
*FBXO32*	Y										[160]
F-Box Protein 32											
*FHL2*	Y										[161]
Four and a Half LIM Domains 2											
*FKTN*	Y										[162]
Fukutin											
*FKRP*	Y										[163]
Fukutin Related Protein											
*FLNC*										Y	[75]
Filamin C											
*FOXD4*	Y										[164]
Forkhead Box D4											
*FSTL5*		Y									[58]
Follistatin Like 5											
*GATAD1*	Y										[165]
GATA Zinc Finger Domain Containing 1											
*HAND1*	Y										[166]
Heart and Neural Crest Derivatives Expressed 1											
*HCG22*	Y										[167]
HLA Complex Group 22											
*HLA-DQB1*	Y										[167]
Major Histocompatibility Complex, Class II, DQ Beta 1											
*HSPB7*	Y										[168]
Heat Shock Protein Family B (Small) Member 7											
*ILK*	Y										[169]
Integrin Linked Kinase											
*JPH2*					Y						[170]
Junctophilin 2											
*JUP*							Y				[171]
Junction Plakoglobin											
*LAMA2*	Y										[172]
Laminin Subunit Alpha 2											
*LAMA4*	Y										[169]
Laminin Subunit Alpha 4											
*LAMP2*	Y									Y	[75,173]
Lysosomal Associated Membrane Protein 2											
*LDB3*	Y				Y						[174,175]
LIM Domain Binding 3											
*LMNA*	Y						Y			Y	[75,176,177]
Lamin A											
*LRRC10*	Y										[178]
Leucine Rich Repeat Containing 10											
*MURC*	Y										[179]
Muscle Related Coiled-Coil Protein											
*MYBPC3*	Y				Y	Y				Y	[24,28,75,180,181,182]
Myosin Binding Protein C, Cardiac											
*MYH6*	Y				Y						[67,183,184]
Myosin Heavy Chain 6											
*MYH7*	Y				Y					Y	[75,185,186]
Myosin Heavy Chain 7											
*MYL2*					Y						[187]
Myosin Light Chain 2											
*MYL3*					Y						[188]
Myosin Light Chain 3											
*MYOZ2*					Y						[189]
Myozenin 2											
*MYPN*	Y										[190]
Myopalladin											
*NEBL*	Y										[191]
Nebulette											
*NEXN*	Y										[192]
Nexilin F-Actin Binding Protein											
*NFKB1*	Y								Y		[95,96]
Nuclear Factor Kappa B Subunit 1											
*NOS3*	Y										[193]
Nitric Oxide Synthase 3											
*OPA3*			Y								[44]
Outer Mitochondrial Membrane Lipid Metabolism Regulator											
*PDE3B*		Y									[58,124]
Phosphodiesterase 3B											
*PDK4*		Y									[56]
Pyruvate Dehydrogenase Kinase 4											
*PKP2*	Y						Y				[84,194]
Plakophilin 2											
*PLEKHM2*	Y										[195]
Pleckstrin Homology and RUN Domain Containing M2											
*PLN*	Y				Y		Y				[196,197,198]
Phospholamban											
*POLG*	Y										[199]
DNA Polymerase Gamma, Catalytic Subunit											
*PRDM16*	Y										[200]
PR/SET Domain 16											
*PRKAG2*					Y						[201]
Protein Kinase AMP-Activated Non-Catalytic Subunit Gamma 2											
*PSEN1*	Y										[201]
Presenilin 1											
*PSEN2*	Y										[202]
Presenilin 2											
*RBM20*	Y										[203]
RNA Binding Motif Protein 20											
*RETN*	Y										[204]
Resistin											
*RMND1*	Y										[205]
Required for Meiotic Nuclear Division 1 Homolog											
*RRAGC*	Y										[206]
Ras Related GTP Binding C											
*RYR2*	Y				Y		Y				[207,208,209]
Ryanodine Receptor 2											
*SCN5A*	Y										[210]
Sodium Voltage-Gated Channel Alpha Subunit 5											
*SDHA*	Y										[211]
Succinate Dehydrogenase Complex Flavoprotein Subunit A											
*SGCD*	Y										[212]
Sarcoglycan Delta											
*STRN*		Y						Y			[57,94]
Striatin											
*SYNE1*	Y										[213]
Spectrin Repeat Containing Nuclear Envelope Protein 1											
*TAZ*	Y										[104]
Tafazzin											
*TBX20*	Y										[214]
T-Box 20											
*TBX5*	Y										[215]
T-Box 5											
*TCAP*	Y				Y					Y	[75,216]
Titin-Cap											
*TGFB3*							Y				[217]
Transforming Growth Factor Beta 3											
*TMEM43*							Y				[218]
Transmembrane Protein 43											
*TMPO*	Y										[219]
Thymopoietin											
*TNNC1*	Y				Y						[118,220]
Troponin C1, Slow Skeletal and Cardiac Type											
*TNNI3*	Y				Y					Y	[75,79,220,221]
Troponin I3, Cardiac Type											
*TNNT2*	Y			Y	Y					Y	[46,75,222,223]
Troponin T2, Cardiac Type											
*TPM1*	Y				Y					Y	[75,183,223]
Tropomyosin 1 (Alpha)											
*TXNRD2*	Y										[224]
Thioredoxin Reductase 2											
*TTN*	Y				Y		Y				[220,225,226]
Titin											
*VCL*	Y				Y						[227,228]
Vinculin											
*ZBTB17*	Y										[229]
Zinc Finger and BTB Domain Containing 17											
